# CEACAM1 as a molecular target in oral cancer

**DOI:** 10.18632/aging.204960

**Published:** 2023-08-16

**Authors:** Sai Ma, Zhonghua Wang, Chao Li, Zhenli Liu, Xuan Zhang, Liheng Li, Feng An, Xiaoli Qiao

**Affiliations:** 1 Department of Stomatology, The First Affiliated Hospital of Hebei North University, Zhangjiakou, Hebei 075000, China; 2 Department of Central Sterile Supply, The First Affiliated Hospital of Hebei North University, Zhangjiakou, Hebei 075000, China

**Keywords:** CEACAM1, molecular target, oral cancer, bioinformatics

## Abstract

Objective: The majority of oral cancer is caused by malignant transformation of squamous cells in surface of the oral mucosa. However, the relationship between CEACAM1 and oral cancer is unclear.

Methods: GSE23558 and GSE25099 profiles were downloaded from gene expression omnibus (GEO). Differentially expressed genes (DEGs) were screened and weighted gene co-expression network analysis (WGCNA) was performed. Construction and analysis of protein-protein interaction (PPI) Network. Gene Ontology (GO) and Kyoto Encyclopedia of Gene and Genome (KEGG), gene set enrichment analysis (GSEA), gene expression heatmap, immune infiltration analysis, comparative toxicogenomics database (CTD) were performed. TargetScan screened miRNAs that regulated central DEGs. Western blotting (WB) experiment was performed.

Results: 1269 DEGs were identified. According to GO analysis, they were mainly enriched in same protein binding, signal receptor binding, cell surface, epithelial cell development. KEGG analysis showed that they were mainly enriched in cancer pathways, PI3K Akt signaling pathway, TNF signaling pathway, NF kappa B signaling pathway, TGF beta signaling pathway. PPI network showed that 11 genes (CDCA8, CCNA2, MELK, KIF2C, CDC45, HMMR, TPX2, CENPF, CDK1, CEP55, CEACAM1) were obtained. Gene expression heatmap showed that CEP55 and MELK were highly expressed in oral cancer samples. CEACAM1 was lowly expressed in oral cancer samples. CEACAM1, CEP55 and MELK were involved in tumor, inflammation, necrosis, and proliferation. Western blotting (WB) showed that CEACAM1 in oral cancer samples was lower than that in normal samples, after CEACAM1 knockdown, it was lower than that in oral cancer samples.

Conclusion: CEACAM1 is lowly expressed in oral cancer, the lower CEACAM1, the worse prognosis.

## INTRODUCTION

Oral cancer is a malignant tumor that occurs in tissues and organs within the oral cavity and usually originates from cells in the oral cavity and can spread to lymph nodes in the neck, other tissues and organs in the head and neck, and even other parts of the body [1, 2]. The age of onset of oral cancer is mainly in the range of 40–70 years, and incidence of oral cancer is higher in developed countries, as well as in parts of Asia and Africa [3]. Oral cancer can occur at various sites within the oral cavity, such as the lip, tongue, and oral mucosa, and has many types, is aggressive, is easy to recur, and early symptoms will be slight pain, discomfort, ulceration, and so on, which can be easily overlooked or misdiagnosed [4]. The clinical manifestations of oral cancer are intraoral pain or discomfort, the presence of prolonged oral ulcers, gingival bleeding or gingival swellings, difficulty chewing and swallowing, intraoral masses or masses, halitosis, and hoarseness or alteration of voice [5]. Oral cancer can be classified into several histological types, which are classified into several differentiation states: well differentiated, moderately differentiated, and poorly differentiated oral cancer can infiltrate adjacent tissues, such as the jaws and neck lymph nodes, infiltrate into surrounding blood vessels, and form new blood vessels. The morphology of oral cancer is characterized by the presence of large nuclei, increased mitotic figures Heterotypic changes such as disorganized cell arrangement [6, 7]. Oral cancer affects the patient’s appetite, and in severe cases undernutrition, as well as masticatory and phonatory abilities, with infections and bleeding [8]. However, the causes of oral cancer are not clear.

Bioinformatics is an interdisciplinary field that involves computer science, mathematics, biology, and statistics. The development of bioinformatics technology has greatly assisted biological research, accelerating the interpretation and understanding of biomolecules such as genomes, proteins, and metabolomes [9]. Bioinformatics technology includes sequence analysis, structure analysis, functional prediction, systems biology, genomics, and proteomics. Bioinformatics technology is constantly evolving, allowing for more efficient and accurate interpretation of biological information. The advantages of bioinformatics technology are mainly reflected in its efficiency, accuracy, visualization, and reproducibility [10, 11]. However, the relationship between CEACAM1 and oral cancer is unclear at present.

This study intended to use bioinformatics to mine core genes between oral cancer and normal tissues, and correlation analyses were performed. Public datasets were utilized to validate role of CEACAM1 in oral cancer. And the basal cell experiment was applied to verify it.

## METHODS

### Oral cancer datasets

In this study, oral cancer datasets GSE23558 and GSE25099 configuration files were downloaded from the GEO (https://www.ncbi.nlm.nih.gov/geo/) generated by GPl6480, GPl5175. GSE23558 including 27 oral cancer and 5 normal samples, GSE25099 including 57 oral cancer and 22 normal samples.

### Screening of differentially expressed genes (DEGs)

Probe aggregation and background correction of merge matrix of GSE23558 and GSE25099 using R package “limma”. *P* value were adjusted using Benjamini-Hochberg method. The fold change (FC) is calculated using false discovery rate (FDR). The cutoff value of DEG is *p* less than 0.05 and FC greater than 1.2. And make a visual representation of the volcano. After that, the differential genes of GSE23558 and GSE25099 were intersected to obtain DEGs.

### Weighted gene co expression network analysis (WGCNA)

First of all, use the gene expression profile of GSE23558 and GSE25099 to calculate Median Absolute Deviation (MAD) of each gene. The good sample gene method of WGCNA in R package was used to remove outlier genes and samples to construct a scale-free co-expression network. We calculated characteristic gene differences of modules, and selected tangent line for module tree view, incorporated part of modules.

### Construction and analysis of protein-protein interaction (PPI) networks

Search Tool for the Retrieval of Interacting Genes (STRING, https://string-db.org/) is a search system for known and predicted PPI. STRING database also contains the predicted results using bioinformatics methods. The differential genes were input into STRING to construct PPI network and predict core genes. PPI network was visualized, core genes are predicted by Cytoscape software (https://cytoscape.org). First of all, we import PPI network into the Cytoscape, and then find module with the best correlation through MCODE. MCC and MNC were used to calculate the best correlated genes. Finally, the list of core genes was obtained after visualization.

### Functional enrichment analysis

Gene Ontology (GO) analysis is a computational method to evaluate gene functions and biological pathways, and it is a key step to endow sequence information with practical biological significance. Kyoto Encyclopedia of Gene and Genome (KEGG) is an online database dedicated to collecting information on genomes, molecular interaction networks, enzyme catalytic pathways, and biochemical products. The genomic information and gene function were linked, and gene function was systematically analyzed. The list of differential genes screened by Venn diagram was input into KEGG rest API obtained latest KEGG Pathway gene annotation. Gene set enrichment results were obtained using R package cluster Profiler.

Metascape (http://metascape.org/) can realize cognition of gene or protein function, and can be visually exported. We used Metascape database to analyze functional enrichment of the above differential gene list and derive it.

### GSEA

GSEA (http://software.broadinstitute.org/gsea/index.jsp) is based on level-specific gene probes that evaluate data from microarrays and is a way to uncover genomic expression data through fundamental knowledge. The samples were divided into oral cancer and normal tissue. 5 is minimum gene set and 5000 is maximum gene set, 1000 resampling times. The whole genome was analyzed by GO and KEGG.

### Gene expression heatmap

We use R-packet heatmap to map expression of core genes found in PPI network in GSE23558 and GSE25099, and to visualize difference of core gene expression between oral cancer and normal tissue samples.

### Immune infiltration analysis

The CIBERSORT (http://CIBERSORT.stanford.edu/) is a very common method for calculating immune cell infiltration. We applied the integrated bioinformatics method, used the CIBERSORT software package to analyze the GSE23558 and GSE25099, and immune cell abundance was estimated by deconvoluting the expression matrix of immune cell subtypes by linear support vector regression principle. At the same time, the samples with sufficient confidence were selected by using confidence *P* < 0.05 as the truncation criterion.

### CTD analysis

CTD (http://ctdbase.org/) is a powerful public database, which predict gene/protein relationships with disease, are used to identify integrated chemical diseases, chemical genes, and gene disease interactions to predict new associations and generate extended networks. We input core gene into CTD, find disease most related to core gene and drew an expression difference radar plot for each gene with Excel.

### Western blotting (WB)

Western blotting is a method of detecting the expression of certain proteins in complex samples according to the specific combination of antigens and antibodies, and can be used for qualitative and semi-quantitative analysis of proteins. Total protein was extracted and its content was determined. After SDS-PAGE electrophoresis and membrane transfer, the protein samples were sealed with 5% skim milk at room temperature for 1 h, and then shaken with Tris Buffered Saline Tween on a shaker at high speed for 5 min and repeated three times. The primary antibody was added, incubated at 4°C overnight, and the secondary antibody was shaken 3 times (5 min/time) at TBST, incubated at room temperature for 1 h, and shaken 3 times (5 min/time) at TBST. The results were analyzed after chemiluminescent solution development.

### The miRNA

TargetScan (http://www.targetscan.org) can predict and analyze miRNA and target genes. Screening of miRNAs regulating central DEGs was performed using TargetScan in this study.

### Availability of data and materials

The datasets generated during and/or analyzed during the current study are available from the corresponding author on reasonable request.

## RESULTS

### Analysis of differentially expressed genes

**Figure 1:**
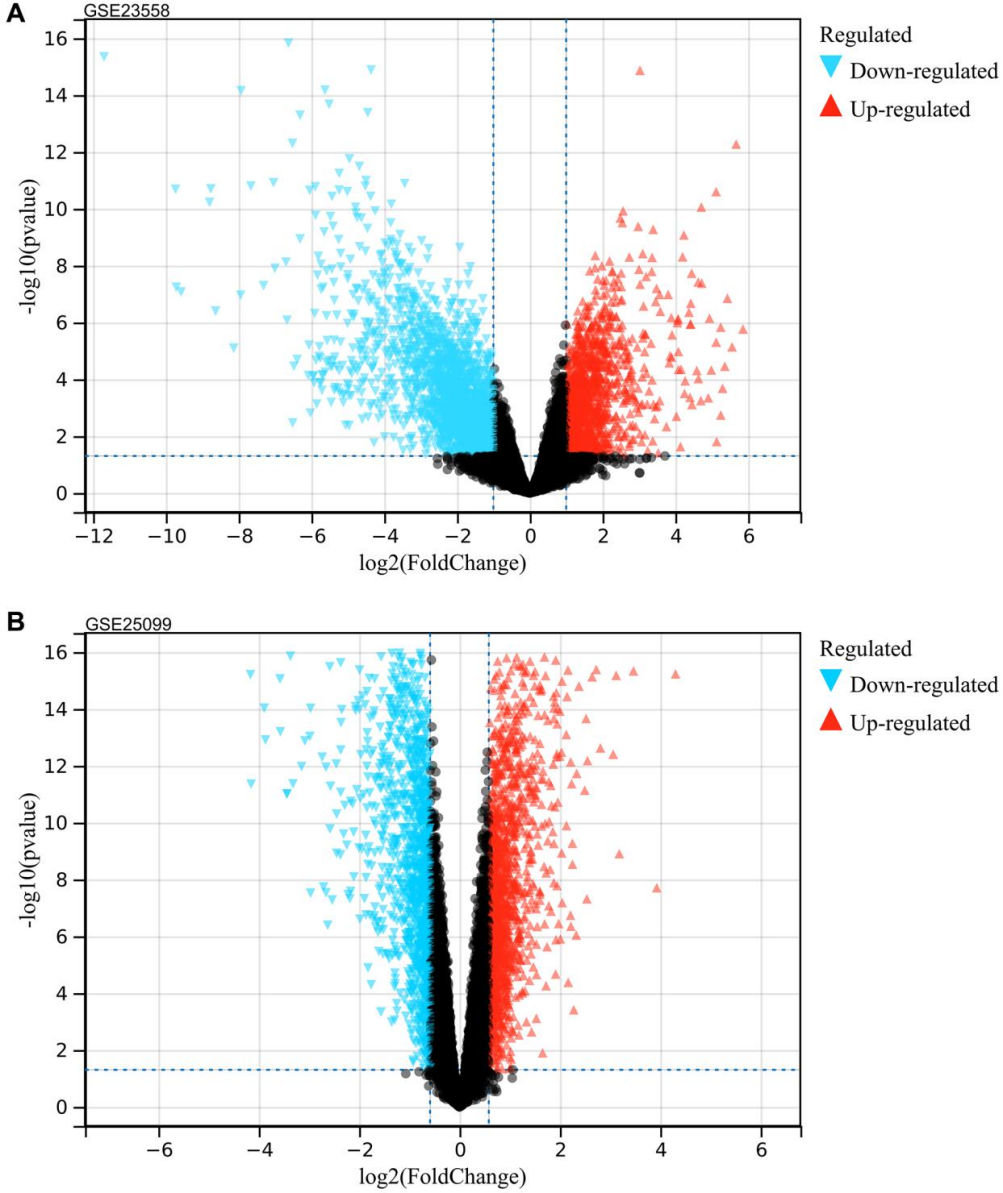
**Analysis of differentially expressed genes.** (**A**) GSE23558 (**B**) GSE25099.

**Figure 2:**
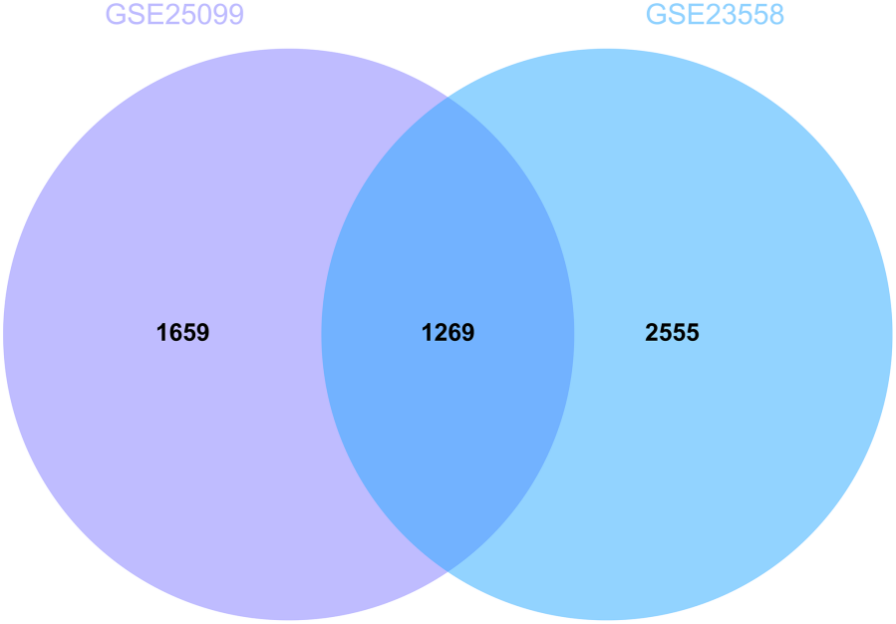
**Analysis of differentially expressed genes.** 1269 DEGs were identified.

Following the set cut-off value, differentially expressed genes were identified according to GSE23558 and GSE25099, respectively (Figure 1A for GSE23558 results and Figure 1B for GSE25099 results), and intersected, 1269 DEGs were identified (Figure 2).

### Functional enrichment analysis

#### DEGs

**Figure 3:**
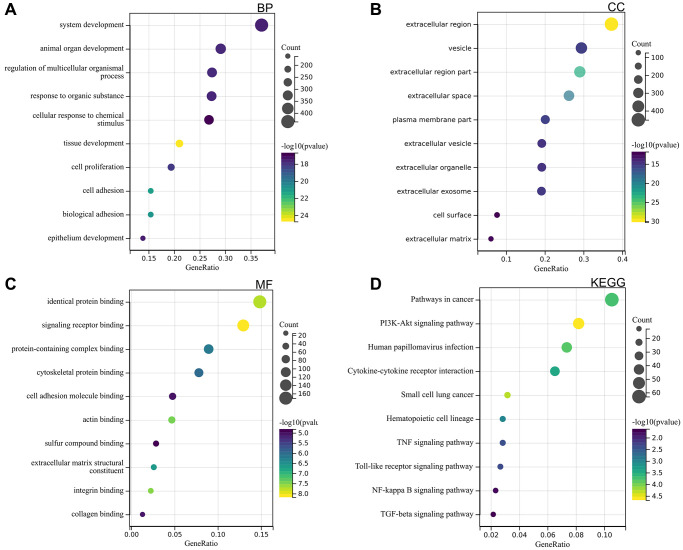
DEGs functional enrichment analysis (**A**) BP (**B**) CC (**C**) MF (**D**) KEGG.

GO and KEGG analyses were performed on DEGs. According to GO analysis, they were mainly enriched in same protein binding, signal receptor binding, cell surface, epithelial cell development (Figure 3A–3C).

KEGG analysis showed that they were mainly enriched in cancer pathways, PI3K Akt signaling pathway, TNF signaling pathway, NF kappa B signaling pathway, TGF beta signaling pathway (Figure 3D).

#### GSEA

**Figure 4:**
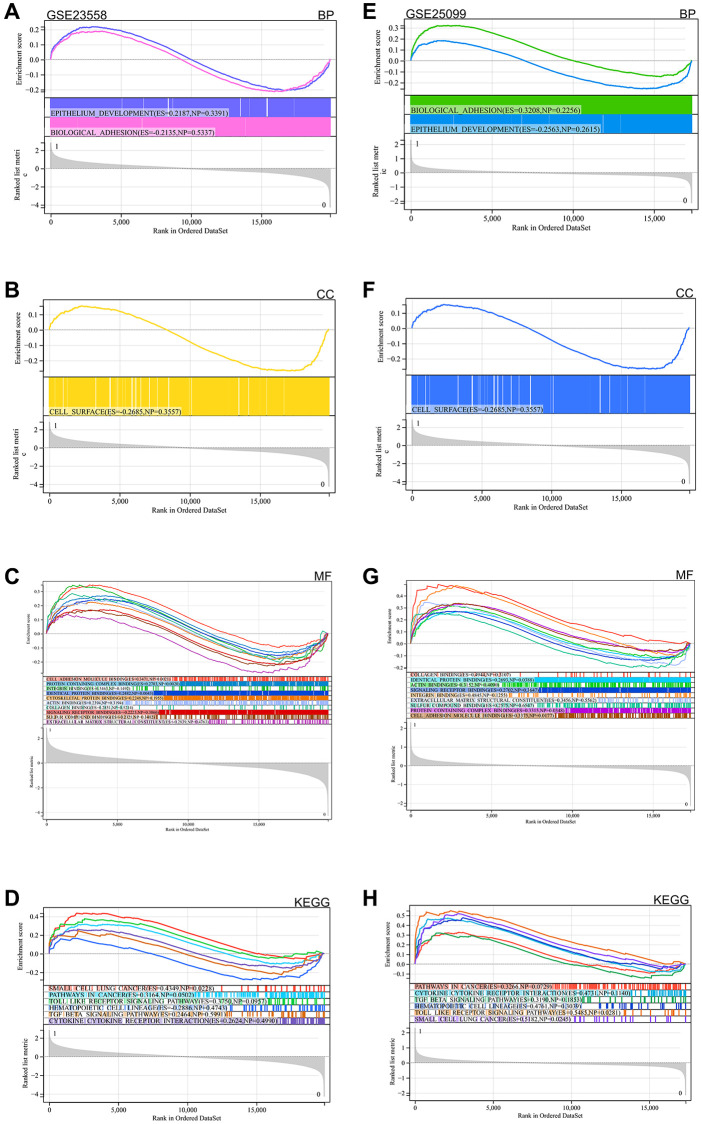
GSEA functional enrichment analysis (**A**–**D**) GSE23558 (**E**–**H**) GSE25099.

A genome-wide GSEA enrichment analysis was performed, aiming to find possible enrichment items in non-differentially expressed genes and validate results for differentially expressed genes, separately for GSE23558 and GSE25099. The intersection of the enriched terms with the GO KEGG enriched terms of the DEGs are mainly enriched in the same protein binding, signaling receptor binding, cell surface, cancer pathways, TGF beta signaling pathway, toll like receptor signaling pathway (Figure 4A–4D for GSE23558 results and Figure 4E–4H for GSE25099 results).

### Metascape enrichment analysis

**Figure 5:**
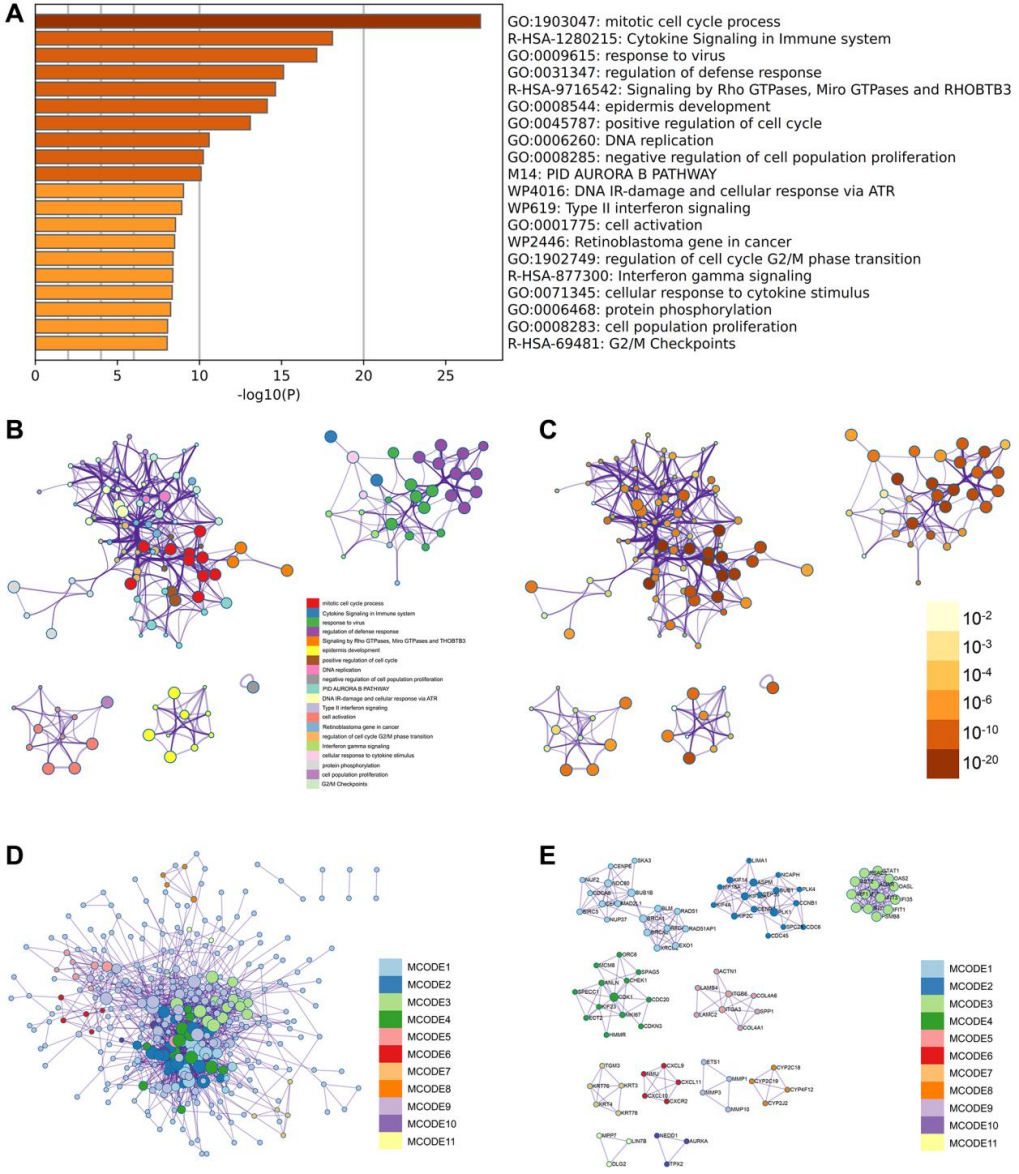
**Metascape enrichment analysis.** (**A**) Positive regulation of cell cycle, PID Aurora B pathway and protein phosphorylation were observed in GO enrichment project (**B**–**E**) the enrichment network colored by enrichment term and *p*-value.

The positive regulation of cell cycle, PID Aurora B pathway, protein phosphorylation (Figure 5A) was observed in GO enrichment items in the enrichment items of metascape, meanwhile we also output the enrichment network colored by enrichment term and *p*-value (Figure 5B–5E) to visualize the association and confidence of each enrichment item.

### WGCNA

**Figure 6:**
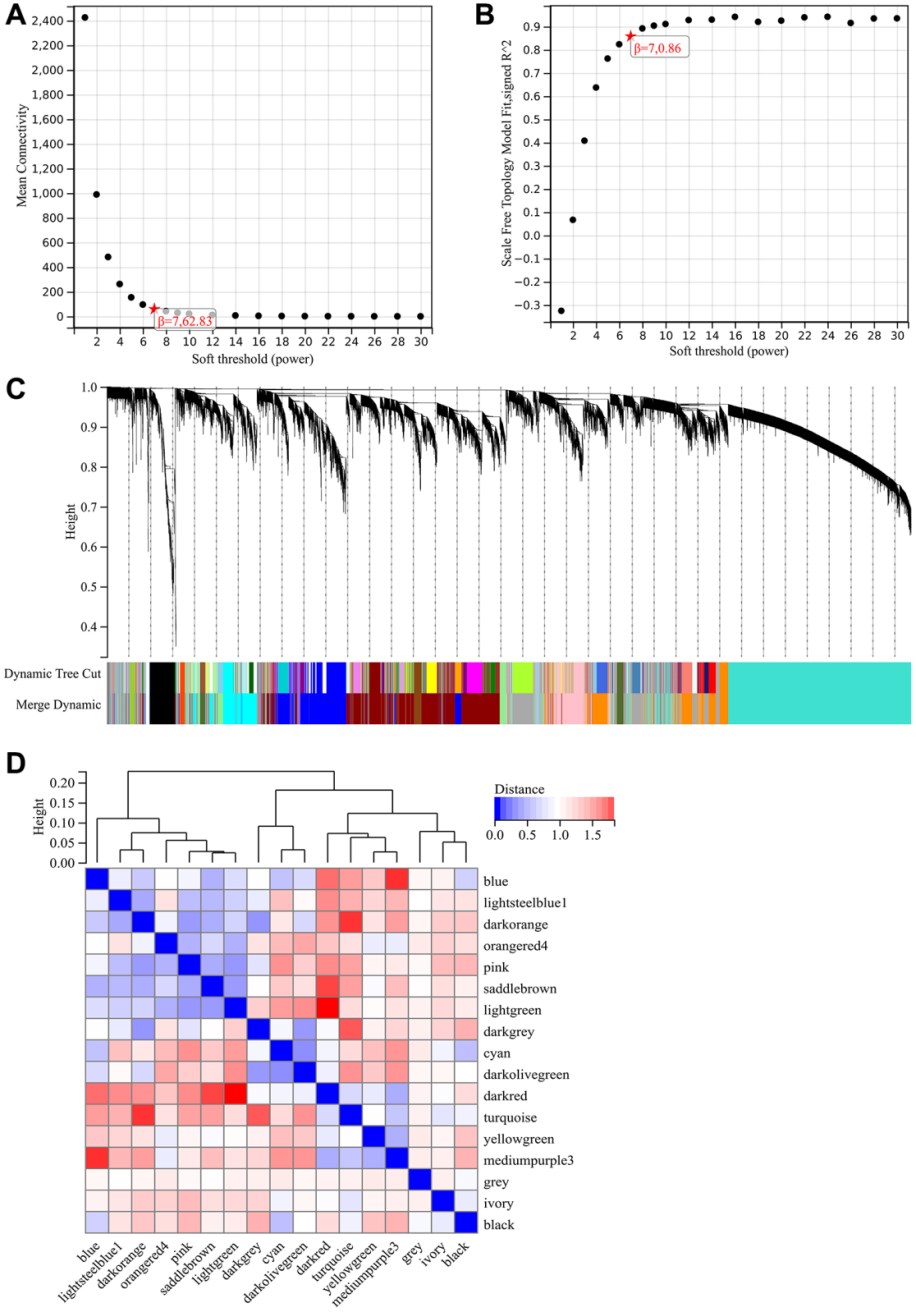
**WGCNA.** (**A**) β = 7,62.38. (**B**) β = 7,0.86. (**C**) Hierarchical clustering trees were constructed for all genes, and significant modules were generated (**D**) The interactions between these modules were analyzed.

**Figure 7:**
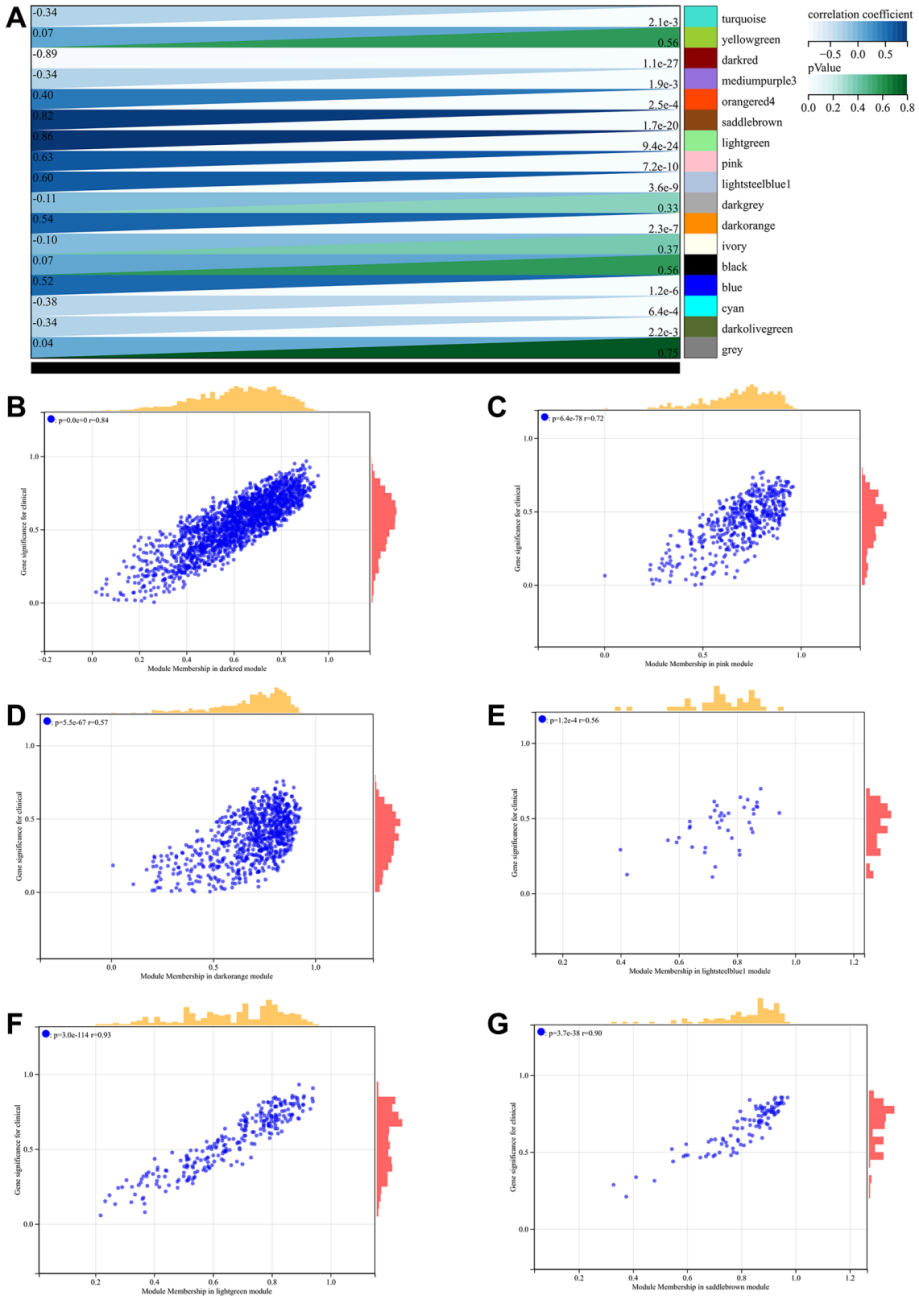
**WGCNA.** (**A**) Generated module to phenotype correlation heatmaps (**B**–**G**) GS to MM correlation scatter plots for the associated hub genes.

The network topology is analyzed and the soft threshold power of WGCNA is set to 5 (Figure 6A, 6B). Hierarchical clustering trees were constructed for all genes, and significant modules were generated, followed by analysis of the interactions between these modules (Figure 6C, 6D). And generated module to phenotype correlation heatmaps (Figure 7A) and GS to MM correlation scatter plots for the associated hub genes (Figure 7B–7G).

### Construction and analysis of protein-protein interaction (PPI) networks

**Figure 8:**
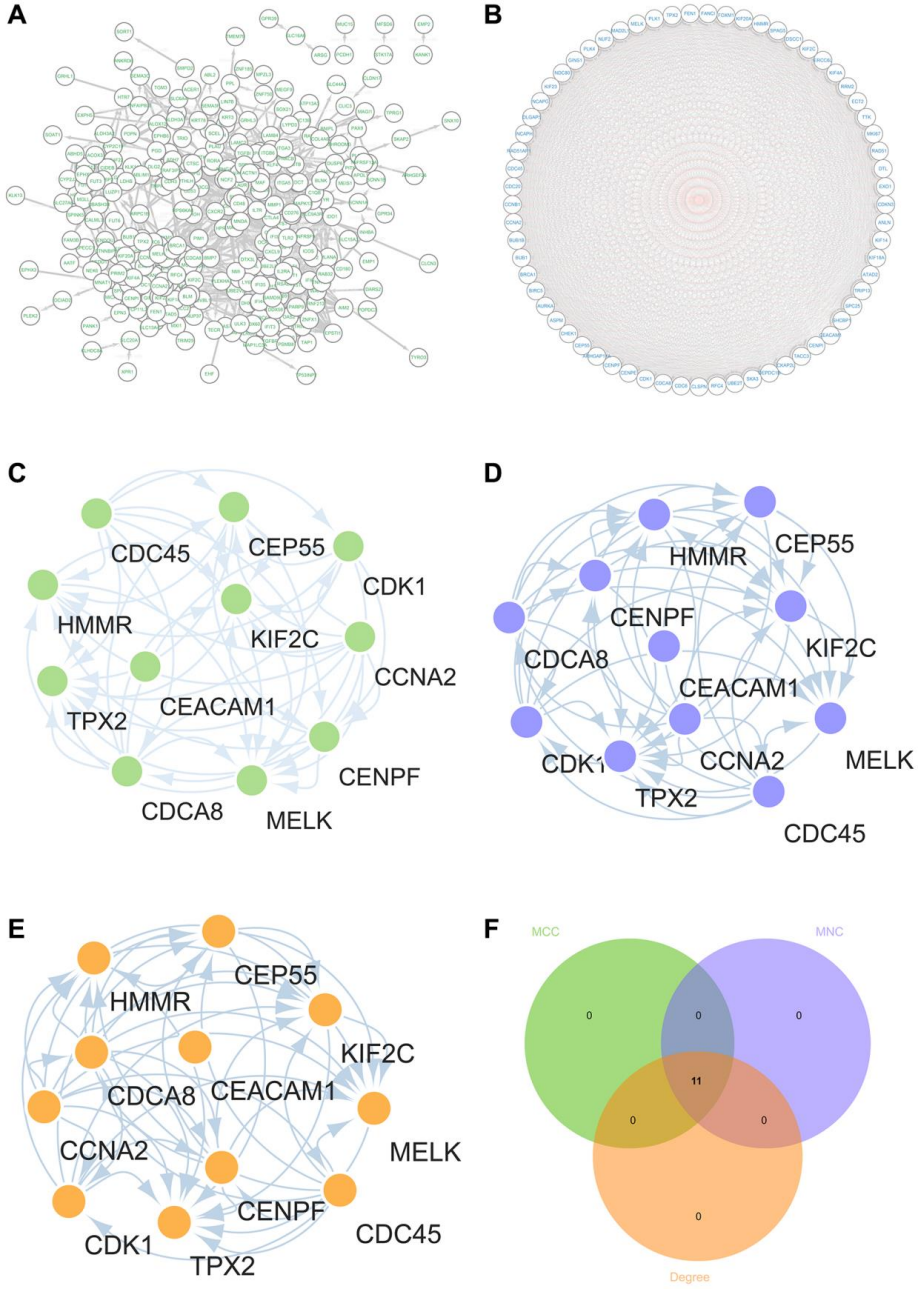
**Construction and analysis of protein-protein interaction (PPI) network.** (**A**) DEGs’s PPI network (**B**) The core gene cluster. (**C**) MCC was used to identify central genes (**D**) MNC was used to identify central genes (**E**) Degree was used to identify central genes (**F**) taking the intersection of Venn diagram.

The PPI network of DEGs was constructed by STRING online database and analyzed by Cytoscape software (Figure 8A), which obtained the core gene clusters (Figure 8B) by using three different algorithms to identify the hub genes (Figure 8C–8E), and taking the intersection of Venn diagram (Figure 8F), 11 core genes (CDCA8, CCNA2, MELK, KIF2C, CDC45, HMMR, TPX2, CENPF, CDK1, CEP55, CEACAM1) were obtained.

Meanwhile, we also output the protein interaction network using the metascape website, and identify the core module to verify the PPI network results in string. Among them (CDCA8, CCNA2, MELK, KIF2C, CDC45, HMMR, TPX2, CENPF, CDK1, CEP55, CEACAM1) genes were identified as core genes.

### Gene expression heatmap

**Figure 9:**
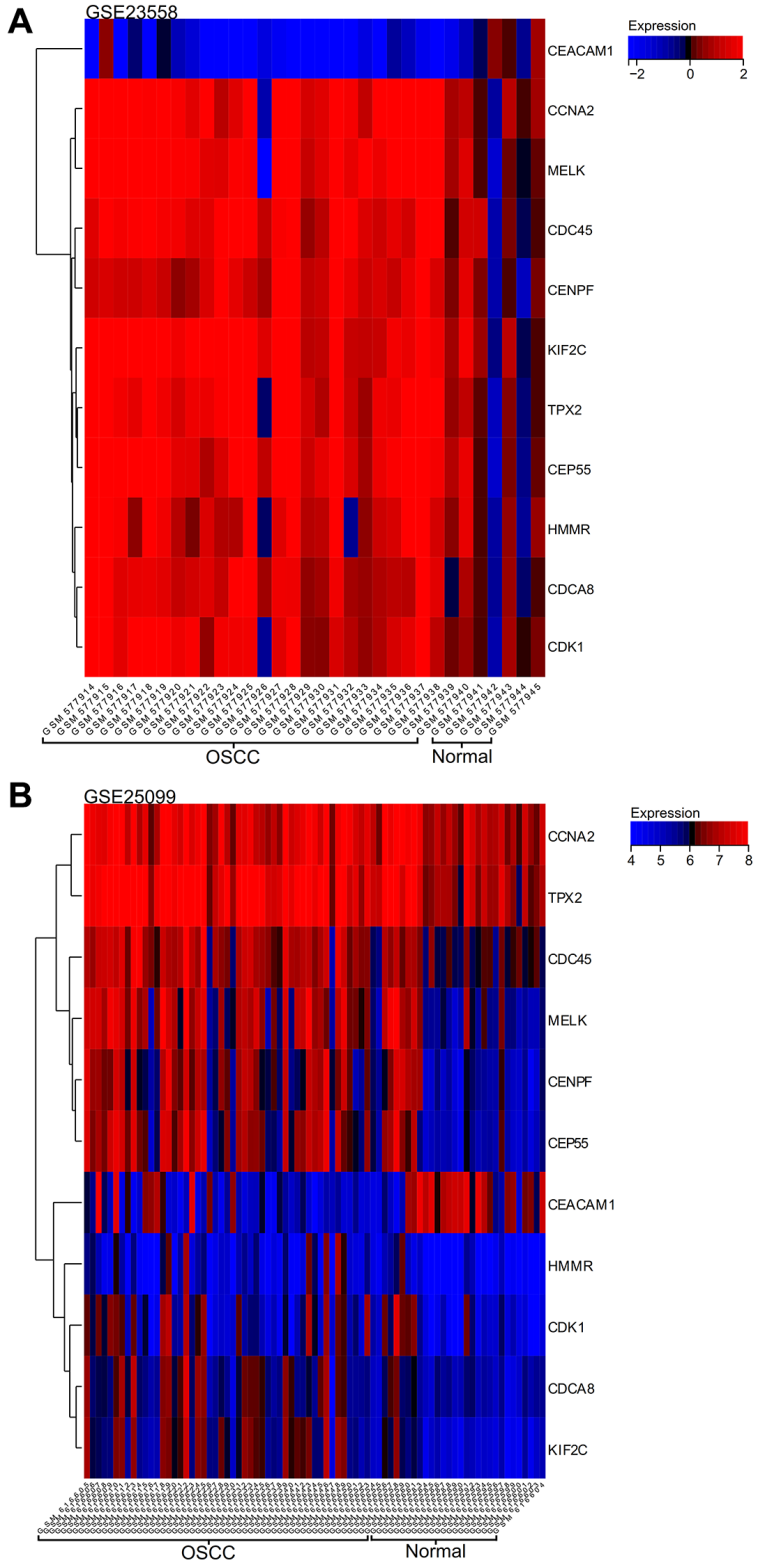
**Gene expression heat map.** (**A**) GSE23558. (**B**) GSE25099.

We visualized expression quantity heatmap of core genes in the samples (Figure 9A for GSE23558 results and Figure 9B for GSE25099 results), and we found that core genes (CEP55, MELK) were highly expressed in oral cancer samples and lowly expressed in normal samples, which may have positive regulatory effects on oral cancer. And the core gene (CEACAM1) was lowly expressed in oral cancer samples and highly expressed in normal samples, which may have a reverse regulatory effect on oral cancer.

### Immune infiltration analysis

**Figure 10:**
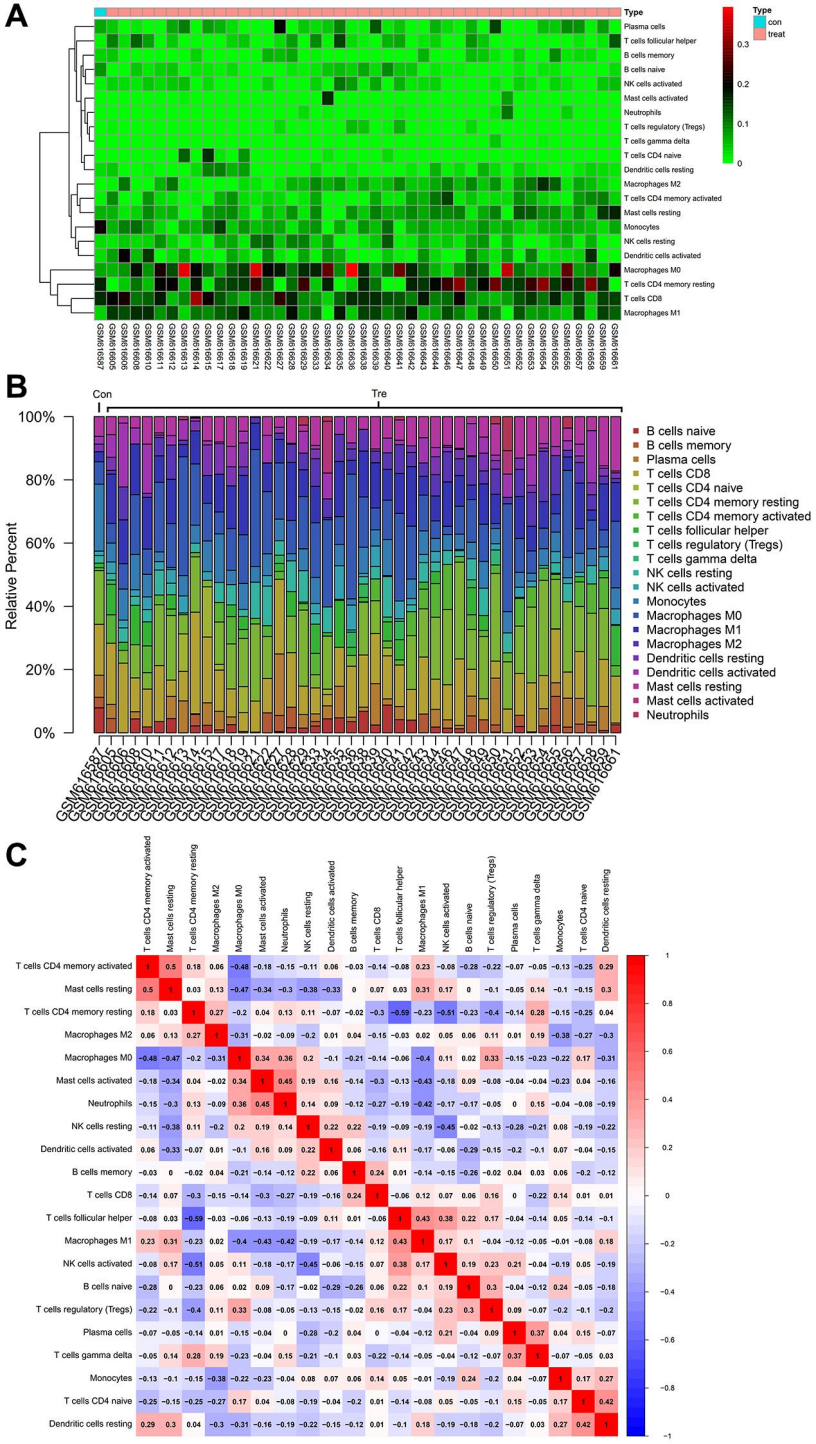
**Immune infiltration analysis.** (**A**) The proportion results of immune cells from the full gene expression matrix (**B**) The immune cell expression heatmap in the dataset (**C**) Diagram of coexpression patterns among immune cell components.

The gene expression matrix of GSE23558 and GSE25099 were analyzed using the cibersort package, at 95% confidence, obtained the proportion results of immune cells from the full gene expression matrix (Figure 10A) and the immune cell expression Heatmap in the dataset (Figure 10B), and also performed correlation analysis on infiltrating immune cells, resulting in a plot of co expression patterns among immune cell components (Figure 10C).

### CTD analysis

**Figure 11:**
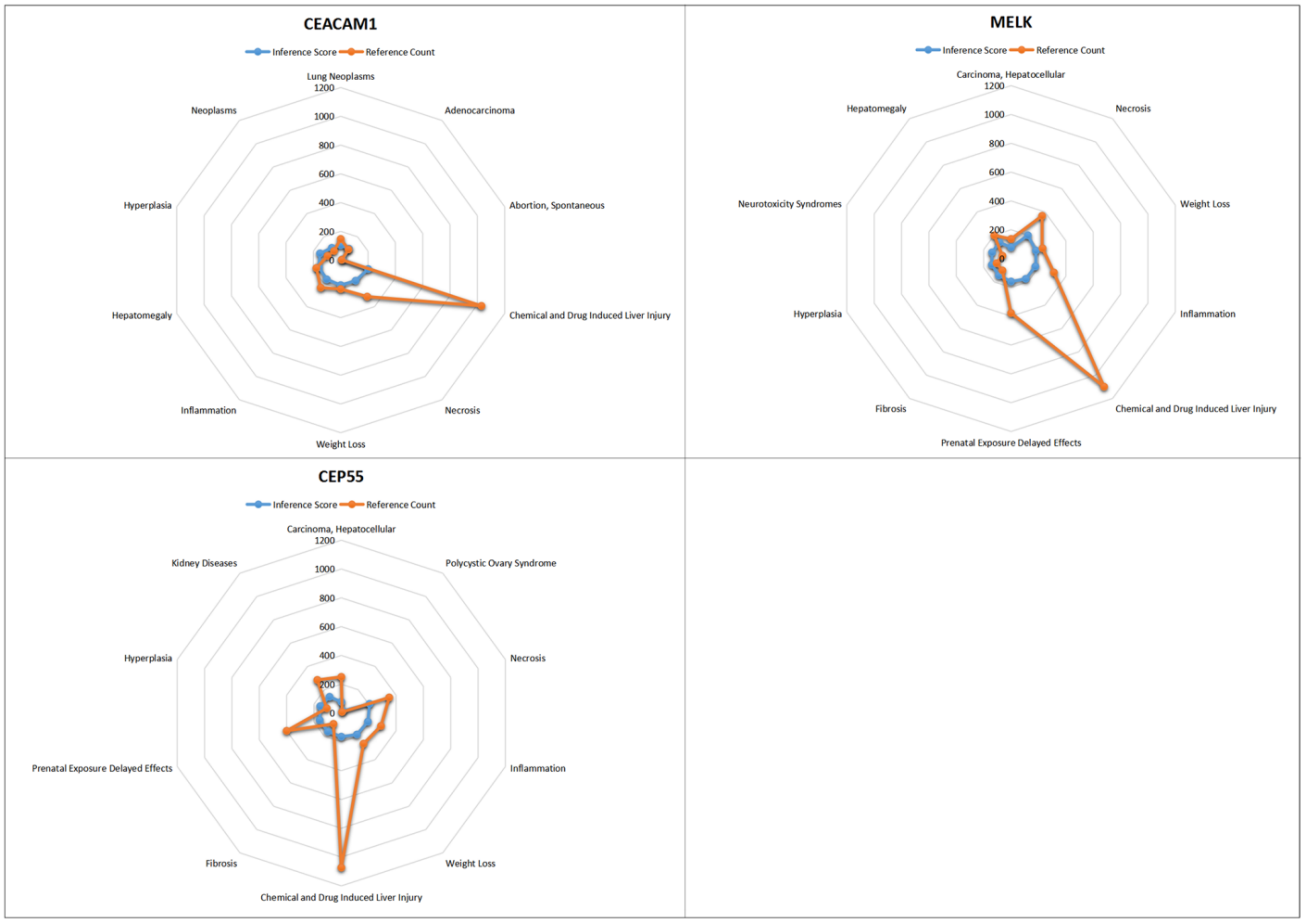
**CTD analysis.** Core genes (CEACAM1, CEP55, MELK) were involved in tumor, inflammation, necrosis, and proliferation.

Core genes was entered into CTD to find diseases related to core genes. Core genes (CEACAM1, CEP55, MELK) and were found to be involved in tumor, inflammation, necrosis, and proliferation (Figure 11).

### Western blotting (WB)

**Figure 12:**
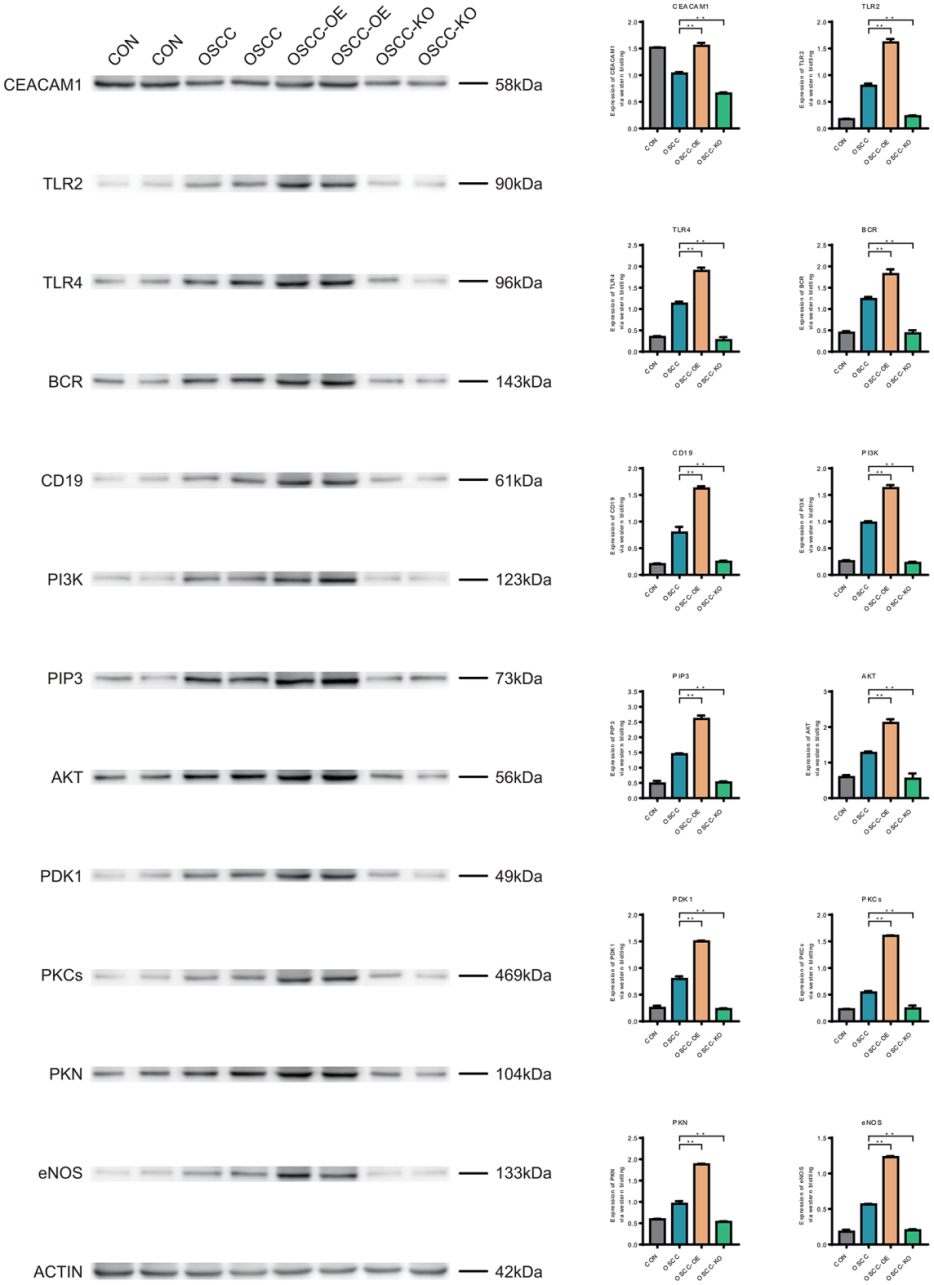
**Western blotting (WB).** CEACAM1, TLR2, TLR4, BCR, CD19, PI3K, PIP3, AKT, PDK1, PKCs, PKN, and eNOS in normal samples, oral cancer samples, overexpression samples and the knockdown samples.

The expression level of CEACAM1 in oral cancer samples was lower than that in normal samples, and after CEACAM1 knockdown, the expression level was lower than that in oral cancer samples and overexpression samples. The expression levels of TLR2, TLR4, BCR, CD19, PI3K, PIP3, AKT, PDK1, PKCs, PKN, and eNOS in oral cancer samples were higher than those in normal samples, and the overexpression samples were higher than those in oral cancer samples, and the expression levels after knockdown were lower than those in oral cancer samples and overexpression samples (Figure 12).

**Figure 13:**
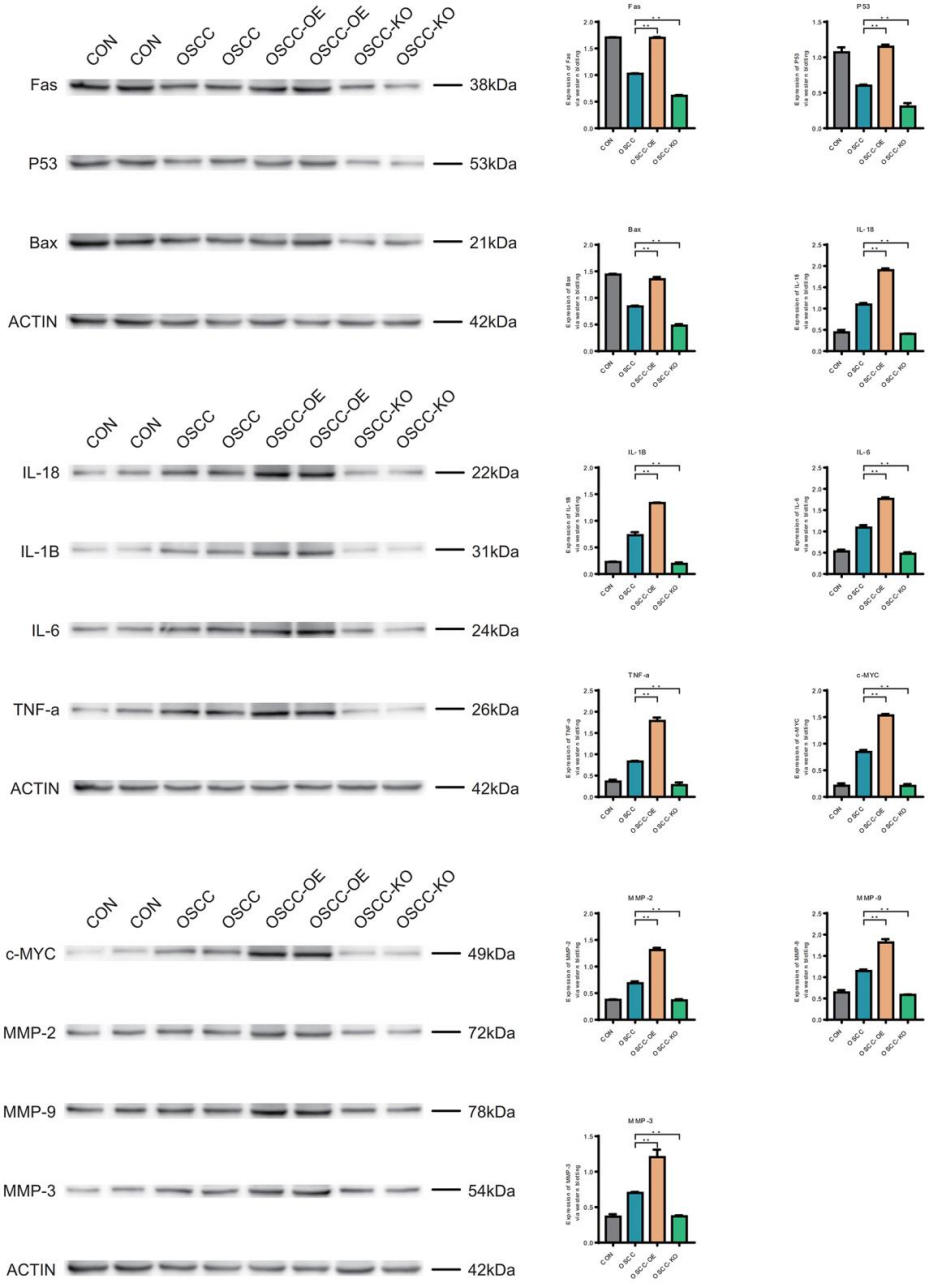
**Western blotting (WB).** FAS, P53, BAX, IL-18, IL-1B, IL-6, TNF-a, c-MYC, MMP-2, MMP-9 and MMP-3 in normal samples, oral cancer samples, overexpression samples and the knockdown samples.

The expression levels of Fas, P53 and BAX in oral cancer samples were lower than those in normal samples, and the expression levels of IL-18, IL-1B, IL-6, TNF-a, c-MYC, MMP-2, MMP-9 and MMP-3 in oral cancer samples were higher than those in normal samples. 11 gene molecules were overexpressed in samples higher than in oral cancer samples, and the expression levels after knockdown were lower than in oral cancer samples and overexpressed samples (Figure 13).

### The miRNAs prediction and functional annotation associated with core genes

The list of hub genes was entered into TargetScan to find relevant miRNAs and improve understanding of gene expression regulation (Table 1). The related miRNAs of CEACAM1 were hsa-mir-30d-5p, hsa-mir-30a-5p, hsa-mir-30b-5p; The related miRNAs of the CEP55 are hsa-mir-144-3p; A related miRNA of the MELK is hsa-mir-802.

**Table 1:** A summary of miRNAs that regulate hub genes.

	**Gene**	**MIRNA**		
1	**CEACAM1**	hsa-miR-30d-5p	hsa-miR-30a-5p	hsa-miR-30b-5p
2	**CEP55**	hsa-miR-144-3p		
3	**MELK**	hsa-miR-802		

## DISCUSSION

Oral cancer is a malignant tumor, and unhealthy lifestyles such as smoking and drinking exacerbate the formation of oral cancer, will lead to discomfort such as oral pain and throat pain, affect the patient’s appetite and nutrient intake, and seriously may also lead to wasting and malnutrition [12]. Chemicals in tobacco can cause DNA damage, gene mutations, and oncogene activation, leading to the occurrence of oral cancer. Long term alcohol consumption is also one of the risk factors for oral cancer, especially in beverages with high alcohol concentrations. Alcohol can damage oral mucosal cells and lead to DNA damage and cell apoptosis. The combination of smoking and drinking can cause greater irritation and damage to the oral mucosa, increasing the risk of oral cancer. Oral cancer if left untreated, cancer cells can continuously spread, endangering the patient’s life, long-term chemotherapy and radiotherapy and other strong treatment means, can cause great physical and psychological stress to the patient, affecting the mental health of the patient [13, 14]. Multiple molecular mechanisms are involved in the occurrence and development of oral cancer, and mutations in several genes, including TP53, CDKN2A, EGFR, and PIK3CA, may lead to unrestricted cancer cell proliferation and growth, inhibit apoptosis and cell cycle regulation [15–18]. Epigenetic changes, such as DNA methylation and histone modification, lead to the abnormal expression of certain genes [19–22]. PI3K/Akt, Wnt/β-Aberrations in several signaling pathways, such as catenin and RAS/Raf/MAPK, lead to uncontrolled cell growth, proliferation and metastasis, which can promote oral cancer development [23, 24]. Chromosomal abnormalities such as chromosomal deletions, rearrangements and number alterations can also lead to the inactivation or overexpression of certain genes, which can affect the proliferation and growth abilities of cancer cells [25, 26]. The occurrence of oral cancer is also related to oral inflammatory reactions. These inflammatory reactions can lead to cell apoptosis, gene mutations, and DNA damage, thereby increasing the risk of cancer cell development. To deeply explore the molecular mechanism of oral cancer, research of targeted drugs is extremely important. The main result of this study is that CEACAM1 is underexpressed in oral cancer, and the lower CEACAM1, the worse the prognosis.

CEACAM1 is an adhesion molecule that belongs to the Carcinoembryonic Antigen (CEA) family. It plays a role in intercellular adhesion and signal transduction. CEACAM1 is distributed on multiple cell surfaces, including white blood cells, liver cells, lung cells, and intestinal epithelial cells. CEACAM1 has various biological functions, including regulating cell proliferation, apoptosis, and adhesion. The main functions of CEACAM1 include inhibiting cell proliferation and metastasis, promoting cell apoptosis, regulating intercellular adhesion and signal transduction, etc. CEACAM1 is also involved in biological processes such as immune cell regulation and antiviral immunity. In oncology, the expression of CEACAM1 is closely related to occurrence and development of tumors. CEACAM1 can also affect tumor immunotherapy by regulating the activation and apoptosis of immune cells. Some studies have shown that CEACAM1 has a dual role in growth and metastasis of tumor cells. In some cases, a decrease in the expression of CEACAM1 may promote tumor proliferation and metastasis; In other cases, overexpression of CEACAM1 may inhibit tumor proliferation and metastasis [27]. Studies have shown that CEACAM1 can have multifaceted effects of immune checkpoint inhibitors and tumor markers and is an attractive target for cancer immunotherapy [28]. It has also been shown that CEACAM1 can regulate the activation induced inhibitory molecule-3 (Tim-3) of T cell immunoglobulin domain and mucin domains involved in tolerance and shown to induce T cell exhaustion in chronic viral infections and cancer [29]. It is therefore speculated that CEACAM1 may play an important role in processes such as cell adhesion and migration in oral cancer. The above literature review is consistent with our results that CEACAM1 is lowly expressed in oral cancer, and the lower CEACAM1, the worse the prognosis.

Although this paper has carried out rigorous bioinformatics analysis, there are still some shortcomings. Animal experiments with overexpression or knockdown of the gene were not performed in this study to further verify the function.

In conclusion, CEACAM1 is underexpressed in oral cancer and may play a significant role in the development of oral cancer through pathways such as cell regulation. CEACAM1 may serve as a molecular target for precision treatment of oral cancer and provide a definite directional basis for the mechanistic study of oral cancer.
